# Transcriptome sequencing revealed the regulation of stem internode length associated with mechanical harvesting in three tea varieties

**DOI:** 10.3389/fpls.2025.1626915

**Published:** 2025-06-30

**Authors:** Xiaozeng Mi, Dahe Qiao, Chun Yang, Juan Chen, Sihui Liang, Yan Guo

**Affiliations:** ^1^ Guizhou Tea Research Institute, Guizhou Academy of Agricultural Sciences, Guiyang, Guizhou, China; ^2^ Guizhou Key Laboratory of Molecular Breeding for Characteristic Horticultural Crops, Guizhou Academy of Agricultural Sciences, Guiyang, Guizhou, China; ^3^ Ministry of Agriculture and Rural Affairs Key Laboratory of Crop Genetic, Resources and Germplasm Innovation in Karst Region, Guizhou Academy of Agricultural Sciences, Guiyang, Guizhou, China

**Keywords:** stem internode length, mechanical harvesting, tea plant, RNA-Seq, WGCNA

## Abstract

**Introduction:**

Internode length of tea plant is one of the key traits affecting the mechanical harvesting effect, but there are relatively few reports on their molecular regulatory.

**Methods:**

Transcriptome sequencing (RNA-seq) was performed on it in order to explain its molecular mechanism. GO, KEGG enrichment and WGCNA were used to identify key genes, and their expression levels in three tea tree varieties were validated by qRT-PCR.

**Result:**

In this study, we measured the internode lengths of the three varieties and found that their internode lengths were ‘*Feiyun*’ > ‘*Qiancha 1*’ > ‘*Longjing 43*’. 10,518 differentially expressed genes were identified through transcriptome sequencing and analysis. GO and KEGG enrichment showed that these differentially expressed genes were mainly enriched in plant hormone signal transduction and DNA-binding transcription factor activity pathways. WGCNA analysis identified two modules significantly correlated with internode length. Combining enrichment analysis with WGCNA results, 28 candidate genes associated with internode length were identified. In addition, it was found that the expression levels of *DELLA* and *GA3ox* were highly expressed in ‘*Longjing 43*’, while *GA2ox*, *WRKY*, and *ERF* were highly expressed in ‘*Feiyun*’, showing significant positive and negative correlations with internode length, respectively.

**Discussion:**

Our results provide candidate genes for studying the molecular mechanism of stem elongation, and provide a theoretical basis for selection machine harvested tea varieties and improvement of mechanical harvesting efficiency.

## 1 Introduction

Tea plant (*Camellia sinensis* (L.) O. Kuntze) is an important economic crop in China (Xia et al., 2020). Tea drinks made by different processing methods are loved by consumers all over the world (Jiang and Wen, 2025; Mozumder et al., 2025). China’s tea plantation area, tea industry, and tea consumption all rank among the top in the world (Liao et al., 2025). In the process of tea production, tea picking is a crucial step in the transition from tea plantation cultivation to product production (Kumar et al., 2023). Improving tea picking efficiency can increase tea production and reduce the labor cost to promote the development of the tea industry (Abhiram, 2024). Tea picking is mainly divided into two types: manual picking and mechanical cutting (Lin et al., 2023). Due to the large scale of tea gardens, concentrated harvesting time, and high cost of manual harvesting, the advantages of mechanical harvesting are becoming increasingly prominent (Han et al., 2019). The traditional harvesting machinery currently widely used is to cut tea trees through gear rotation to obtain fresh tea leaves. Because the mechanical picking needs to cut the tea tree (Aaqil et al., 2023), there are certain requirement traits for the tree shape, stem internode length, and leaf attachment angle of tea plants (You et al., 2017). At present, there are various varieties of tea plans, but not all are suitable for mechanical harvesting, which is closely related to their various traits. It is particularly important to underlying the molecular mechanisms of tea varieties that are more suitable for machine harvesting traits in order to identify or cultivate them. For example, leaf droop is an important trait in tea machine harvesting. A study has found that CsBES1.2 can regulate the expression of *CsEXL3* and thus regulate leaf droop (Liu et al., 2024). Afterwards, they identified a nucleotide variation in the promoter region of the *CsTPR* gene through whole genome association analysis, which regulates tea leaf droop (Liu et al., 2025).

The internode length of tea plant stems is one of the important traits that affect the mechanical harvesting effect (Luo et al., 2023). Generally speaking, longer stem length can increase the distance between buds and leaves, reduce damage to adjacent leaves during mechanical harvesting, and maintain leaf integrity (Pathiranage et al., 2023). Therefore, tea varieties with longer internodes are more suitable for mechanized harvesting. The length of stem internodes varies among different tea varieties, and this difference is caused by the different genetic backgrounds of tea varieties, which are fundamentally the result of gene regulation. *GhSBI1* gene was found to inhibit the elongation of internode xylem cells and regulate the branch internode elongation in cotton (Zhong et al., 2024). RIN1 has been found to regulate the flowering and maturation stages of soybeans, and knocking out RIN1 significantly shortens the inter segmental distance (Li et al., 2023b). Furthermore, Through the correlation analysis between stem internode length and hormone content in 7 tea varieties, it was found that GA_3_ may be an important factor regulating tea plant stem elongation (Luo et al., 2023). The internode length of tea plant stems is an important trait affecting mechanical tea harvesting, but there is still relatively little research on its molecular regulation mechanism.

Plant hormones play an important regulatory role in plant growth and development, and gibberellin (GA), as one of the six major categories of plants, have been proven to be involved in regulating stem elongation in various plants (Jing et al., 2024; Shi et al., 2024). There have also been many advances in the study of the molecular mechanisms of gibberellin metabolism pathway genes and receptor proteins regulating stem elongation. For example, Stephen Pearce’s team found that the *GA3OX3* and *GA1OX1* genes in the GA pathway have a role in affecting GA levels, and demonstrated through mutation experience that these two genes have promoting and inhibitory effects on wheat stem elongation, respectively (Phillips et al., 2025). Chen et al. identified the *GA2ox* family in peach and found that they have a regulatory effect on gibberellin. They used tobacco transformation systems to verify that four *GA2ox* genes have the effect of shortening stem length (Cheng et al., 2021). In rice, ACE1 and DEC1 can act together with GA as promoting and inhibiting factors, respectively, to regulate the growth of internodes (Nagai et al., 2020). DELLA protein is a core negative regulatory factor in the GA signaling pathway, and studies have shown that it can regulate the expression of GA pathway genes through chromatin modification (Li et al., 2023a).

Transcription factors are important protein that regulates gene transcription and expression. They can regulate plant growth and development by modulating the expression of downstream genes (Jiao et al., 2024; Yu et al., 2025). Some studies have revealed the role of transcription factors in regulating stem development and cell elongation. GhMYB4 was found to regulate the expression of Lipid transfer protein (*GhLTP4*) and sucrose transporter (*GhSWEET12*) in cotton, and regulate the elongation of its fiber cells (Duan et al., 2025). The *WRKY* gene has been found to interact with plant hormone signaling pathways such as gibberellin and brassinosteroid to regulate internode length in plants (Wang et al., 2023).AtERF11 has been found to promote the expression of *GA3ox1* and *GA20ox* in the GA synthesis pathway, thereby affecting GA synthesis and regulating the elongation of *Arabidopsis* internodes (Zhou et al., 2016). Subsequently, another study discovered that the variation of TaERF caused wheat dwarfism phenotype and demonstrated the role of this gene in wheat stems elongation (Li et al., 2025).

The correlation between stem internode length and tea plant varieties is influenced by their genetic background. Therefore, this study selected three tea plant varieties with different internode lengths for transcriptome sequencing to identify genes related to stem length and reveal their regulatory mechanisms. Through differentially expression gene and enrichment analysis, it was found that signal transduction and transcription factor related genes are involved in the regulation of stem elongation. Based on the results of WGCNA and enrichment analysis, 28 key genes positively and negatively correlated with stem length were identified. And the expression patterns of 9 genes, including *GA2ox*, *GA3ox*, *DELLA*, and *WRKY* transcription factor, were validated by qRT-PCR. This study provides candidate genes for studying the molecular mechanism of tea plant stem elongation from the perspectives of hormones and transcriptional regulation, deepening our understanding of tea plant stem development and aiding in the screening of suitable machine harvested varieties.

## 2 Materials and methods

### 2.1 Plant materials

**Figure 1:**
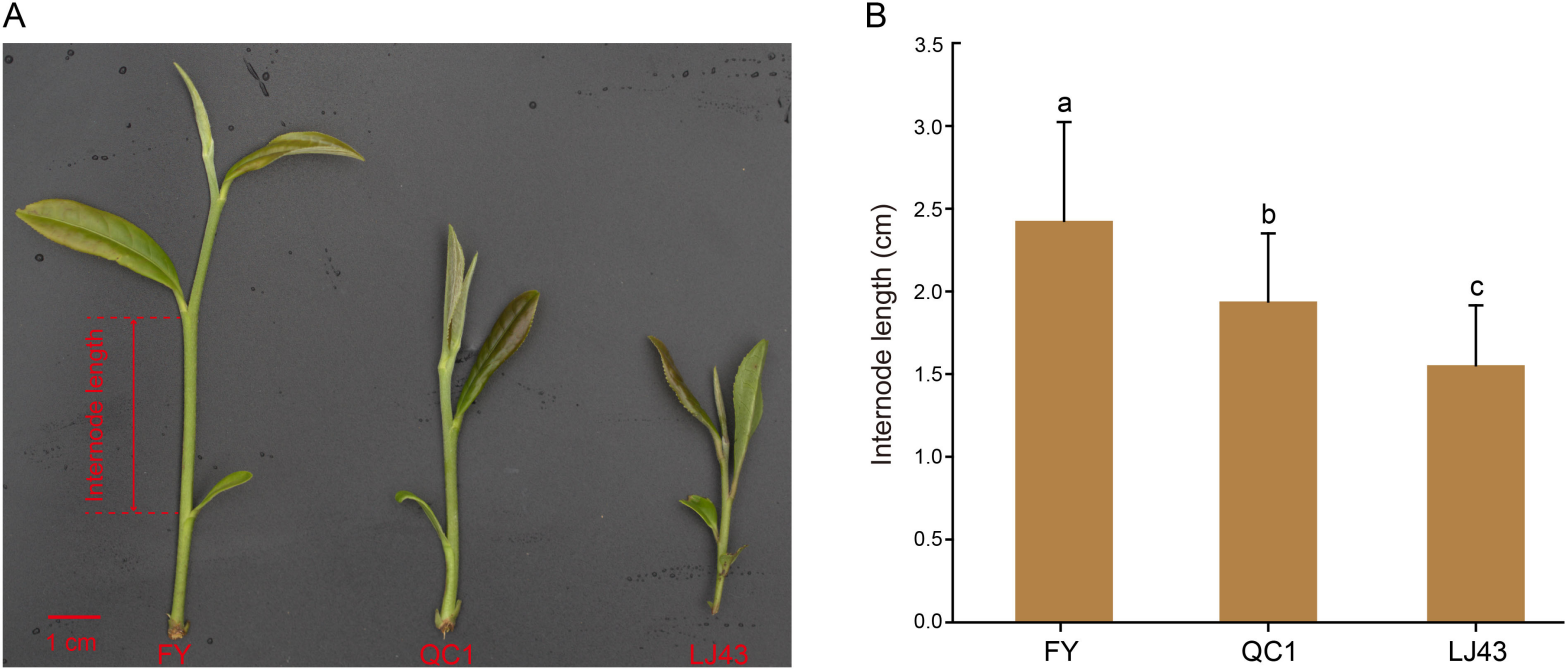
The stem internode length of three tea varieties, namely *Feiyun*, *Qiancha 1*, and *Longjing 43*. **(A)** Pictures of stem internodes of three varieties. The internode length represents the length from the third leaf to the fish leaf, and the length of the red line segment in the bottom left corner represents 1 centimeter; **(B)** The statistical results of internode length for three varieties. Lowercase letters indicate a significance level of P < 0.05.

Three tea varieties, *Feiyun* (FY), *Qiancha 1* (QC1) and *Longjing 43* (LJ43) were planted in tea germplasms in the Germplasm Tea Repository of the Guizhou Tea Research Institute located in Meitan County (N27°45’, E107°29’), Zunyi City, Guizhou Province, China. The reason for choosing these three varieties is that significant differences in stem length were found in the investigation of their stem length. In the spring of 2024, when the tea plants reached the stage of one bud and two leaves, stem tissues of the three tea plant varieties were sampled. The stem tissue between the second and fish leaves of these varieties was used as internode samples (**Figure 1A**), and three biological replicates were collected for each group. All samples were frozen in liquid nitrogen and stored in dry ice. They were brought back to the laboratory and stored at -80°C until use.

### 2.2 Statistics of internode length

When FY, QC1, and LJ43 reach the stage of one bud and two leaves, take 15 branches each from bud to fish leaf as the samples. The representative samples of the three varieties were photographed and the differences in internode length were identified according to their phenotypes. In addition, using a ruler to accurately measure the internode length between the second leaf and the fish leaf of all samples. Record and statistically analyze the measurement results of all samples from three groups, with accuracy rounded to one decimal place.

### 2.3 RNA extraction and sequencing

Nine stem tissues from three varieties were ground in liquid nitrogen and total RNA was extracted using RNAprep Pure Plant Plus Kit (cat DP441, Tiangen, China), according to the manufacturer’s protocol. The quality and quantity of each RNA extract was determined using automated electrophoresis system (Fragment Analyzer 5400) and Qubit fluorescence quantitative analyzer. The qualified RNA was reverse-transcribed and the chain specific library was built for transcriptome sequencing in Lianchuan Biotechnology Co., LTD. Paired-end 150-bp sequencing raw reads were obtained. Clean reads were generated from next-generation sequencing data after removing adapter and primer sequences and low-quality reads using Cutadapt (Martin, 2011). High-quality clean reads were used for subsequent analyses. The raw RNA-seq data are publicly available at the Genome Sequence Archive database of China National Center for Bioinformation (https://ngdc.cncb.ac.cn/gsa) under project accession number PRJCA036838.

### 2.4 Assembly and analysis of transcriptome

Splice-site and exon information were extracted from GTF file with *Qiancha 1* genome (Qiao et al., 2025) annotation using Python scripts in HISAT2 (v.2.2.1) software. Then constructing a reference genome sequence index using the -build parameter of HISAT2 software. All clean data were mapped to the tea plant reference genome using HISAT2 with default parameter to generate SAM files. Samtools software was used to convert Sam files into BAM files (Ramirez-Gonzalez et al., 2012). Transcripts were assembled using the BAM files by StringTie software (v.2.2.1) with default settings (Pertea et al., 2016). To quantify the transcript expression level, the fragments per kilobase of per million mapped fragments (FPKM) were calculated by StringTie. To ensure the reliability of the assembly results, if a transcript has FPKM values less than one in three biological replicates of one sample, it will be removed and the remaining transcript will be considered to exist in the sample. The FPKM of the expressed genes were extracted in each sample and formed a table. The Non-redundant (Nr), Kyoto Encyclopedia of Genes and Genomes (KEGG), and Gene Ontology (GO) annotation information of the reference genome were downloaded for further analysis of the functions of these genes.

### 2.5 Differential expression and enrichment analysis

DEGseq package from R software (v4.2.2) was used to calculate differentially expressed genes (DEGs) based on their FPKM values in different groups (Wang et al., 2010). If the fold-change > 2 and q-values < 0.05 of a gene in two sets of samples, we define this gene as DEG. All DEGs between the two groups were statistically analyzed after removing redundancy and compared using a Venn diagram. TBtools software (Chen et al., 2023) was used for GO and KEGG analysis by their background files and DEGs. GO terms with corrected P < 0.01 were considered significantly enriched and visualizing the enrichment result by t R software.

### 2.6 Weighted gene co-expression network analysis

The stem internode length and expression of DEGs were used for co-expression analysis by the WGCNA package of R software (Pei et al., 2017). Cluster analysis is performed on DEGs, and the weighted values of correlation coefficients are calculated to make the genes in the network follow a scale-free network distribution. Selecting appropriate power values and constructing a one-step network to divide differentially expressed genes into different modules. The detailed parameters are as follow: power = 24, TOMType = unsigned, and branch merge cut height = 0.25. Finally, the constructed gene network and internode length were correlated to identify the gene modules significantly related to the trait.

### 2.7 Quantitative real-time PCR

Total RNA was synthesized into the first strand cDNA using the PrimeScript RT Reagent Kit (cat RR036A, Takara, Japan) using the manufacturer’s protocols. The cDNA of all samples was adjusted to a consistent concentration. The 10ul PCR amplification includes 5 μ L of TB Green (cat RR820A, Takara, Japan), 0.2 μ L of upstream and downstream primers, 1.5 μ L cDNA template, and 3.1 μ L of sterile water, and thermal cycling is performed using a two-step method. All reactions were run in technical triplicates and the CsActin gene was selected as the internal control (Mi et al., 2025). Relative expression of selected genes were analyzed using the 2^-△Ct^ method. All primers used in qRT-PCR were listed in **Supplementary Table S1**.

## 3 Results

### 3.1 Differences in internode length among three varieties

In order to observe the differences in stem internode length among these three tea plant varieties, we measured the internode length from the second leaf to the fish leaf during their one bud and two leaf stages (**Figure 1A**). The results showed that FY had the longest internode length, followed by QC1 and LJ43, with LJ43 having the shortest internode length. The statistical results of measuring 15 internode lengths of each of the three varieties were consistent with the photographic results. The internode length of stem in FY, QC1 and LJ43 showed significant difference at p < 0.05 (**Figure 1B**). FY internode length ranges from 1.8 to 3.8 cm, with an average length of 2.4 cm; QC1 internode length ranges from 1.4 to 2.6 cm, with an average length of 1.9 cm; LJ43 has the shortest internode length, ranging from 1.1 to 2.3 cm with an average length of 1.6 cm (**Figure 1B**; **Supplementary Table S2**). The internode length of FY is significantly greater than that of QC1 and LJ43, and there is also a difference between QC1 and LJ43, but less than the difference between FY and them.

### 3.2 DEGs analysis of stem internode tissues in three varieties

**Figure 2:**
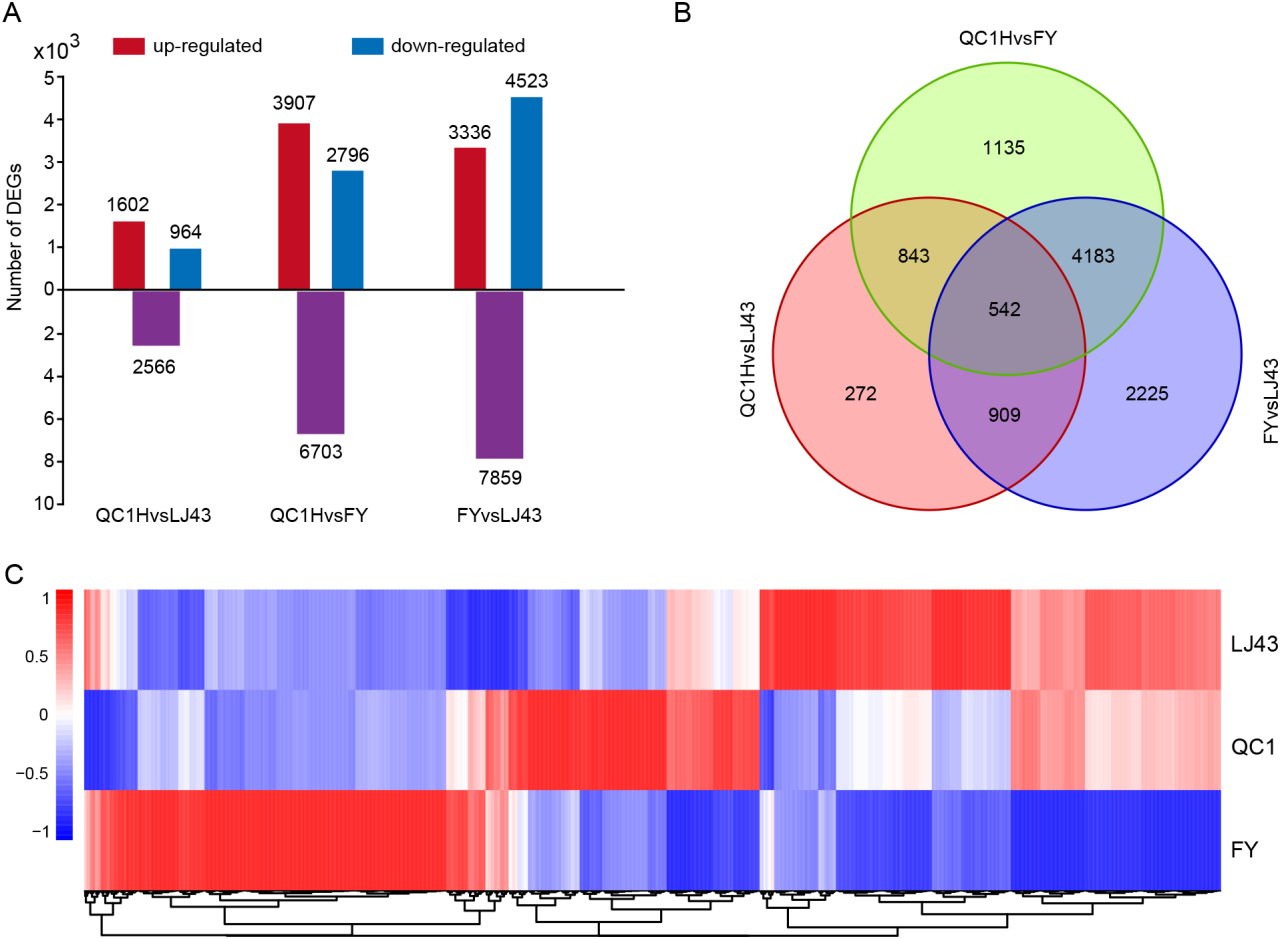
Differentially expressed gene analysis of three tea varieties. **(A)** Statistics on the number of DEGs. Purple, red, and blue represent the total number of DEGs, upregulated genes, and downregulated genes, respectively; **(B)** Venn diagram analysis of DEGs in the three groups; **(C)** Heat map analysis of the expression levels of DEGs. Cluster DEGs according to their expression patterns in three varieties. Blue and red represent low and high expression levels, respectively.

To identify potential genes regulating stem length, we performed RNA-seq on stem tissues of FY, QC1, and LJ43. RNA-seq obtained a total of 48.85 Gb of raw data (range 5.32 ~ 6.31 Gb), Q30 was higher than 96%, GC content was about 44% (**Supplementary Table S3**). The DEGs in the three groups were analyzed, and a total of 10,518 DEGs were identified. Among them, there are 2566 DEGs including 1602 upregulated and 964 downregulated genes in QC1 vs FY, 6703 DEGs including 3907 upregulated and 2796 downregulated genes in QC1 vs LJ43, and 7859 DEGs including 3336 upregulated and 4523 downregulated genes in FY vs LJ43 (**Figure 2A**). We found that the two varieties with the largest internode length difference (FY and LJ43) also had the largest number of DEGs, while QC1H vs FY had the smallest number of DEGs. Furthermore, we performed a Venn diagram analysis of the DEGs in the three comparison groups. The result shows that only 542 of the 10,518 DEGs were differentially expressed in all three comparison groups. 1135, 272, and 2225 DEGs were identified individually only in groups QC1H vs LJ43, QC1 vs FY, and FY vs LJ43 (**Figure 2B**).

Heat maps were used to analyze the expression patterns of DEGs in the three varieties. According to their expression patterns, they can be classified into three categories (**Figure 2C**). The expression level of the first type of DEGs are highest in FY and relatively low in QC1 and LJ43; The expression level of the second type of DEGs are highest in QC1 and relatively low in FY and LJ43; The DEGs in the third category are highest in LJ43, while they are relatively low in FY and QC1. In addition, some DEGs in the first category showed a gradually decreasing trend in FY, QC1, and LJ43, while some DEGs in the third category showed a gradually increasing trend in FY, QC1, and LJ43. The expression trends of these two differentially expressed genes show the same or opposite relationship with the internode length of their stems, and may play a positive or negative regulatory role in the internode length.

### 3.3 Enrichment analysis of DEGs

**Figure 3:**
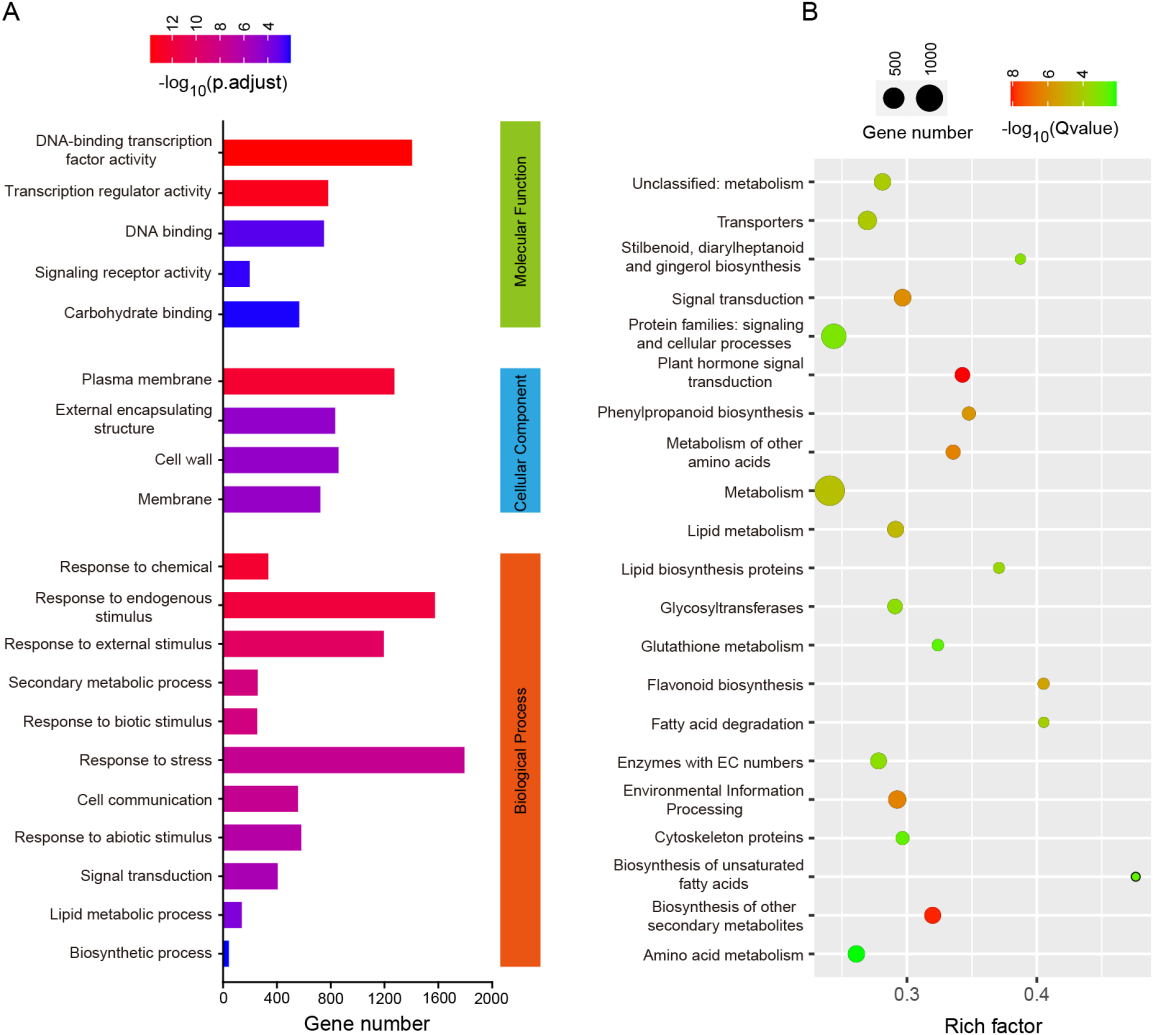
Enrichment analysis of DEGs. **(A)** GO Enrichment Pathway analysis with Q < 0.01. The coordinate values represent the number of genes in the pathway, and the column colors from blue to red indicate the magnitude of enriched Q values; **(B)** KEGG Enrichment Pathway analysis with Q < 0.01. The size and color of the circle represent the number of genes in the pathway and the enriched Q-value, respectively.

To further investigate the functions of these DEGs, we conducted GO and KEGG enrichment analyses on them (**Figure 3**). The GO and KEGG enrichment pathways with p-values less than 0.01 after correction are displayed through bar charts and bubble plots. There are 5, 4, and 11 pathways for DEGs genes in the molecular function, cellular components, and biological processes modules of GO analysis. In molecular function, DEGs are mainly enriched in DNA-binding transcription factor activity, transcription regulator activity and DNA binding. In biological processes, DEGs are mainly enriched in signal transduction and biosynthetic process. KEGG enrichment analysis found that DEGs were mainly enriched in plant hormone signal transduction, signal transduction and flavonoid biosynthesis. It is worth noting that both KEGG and GO analyses enriched signal transduction pathways, indicating that hormone signals or signal receptor proteins may play an important role in regulating internode length. In addition, the regulation of transcription factors is also a major pathway for enrichment.

### 3.4 Analysis of genes associated with internode length

**Figure 4:**
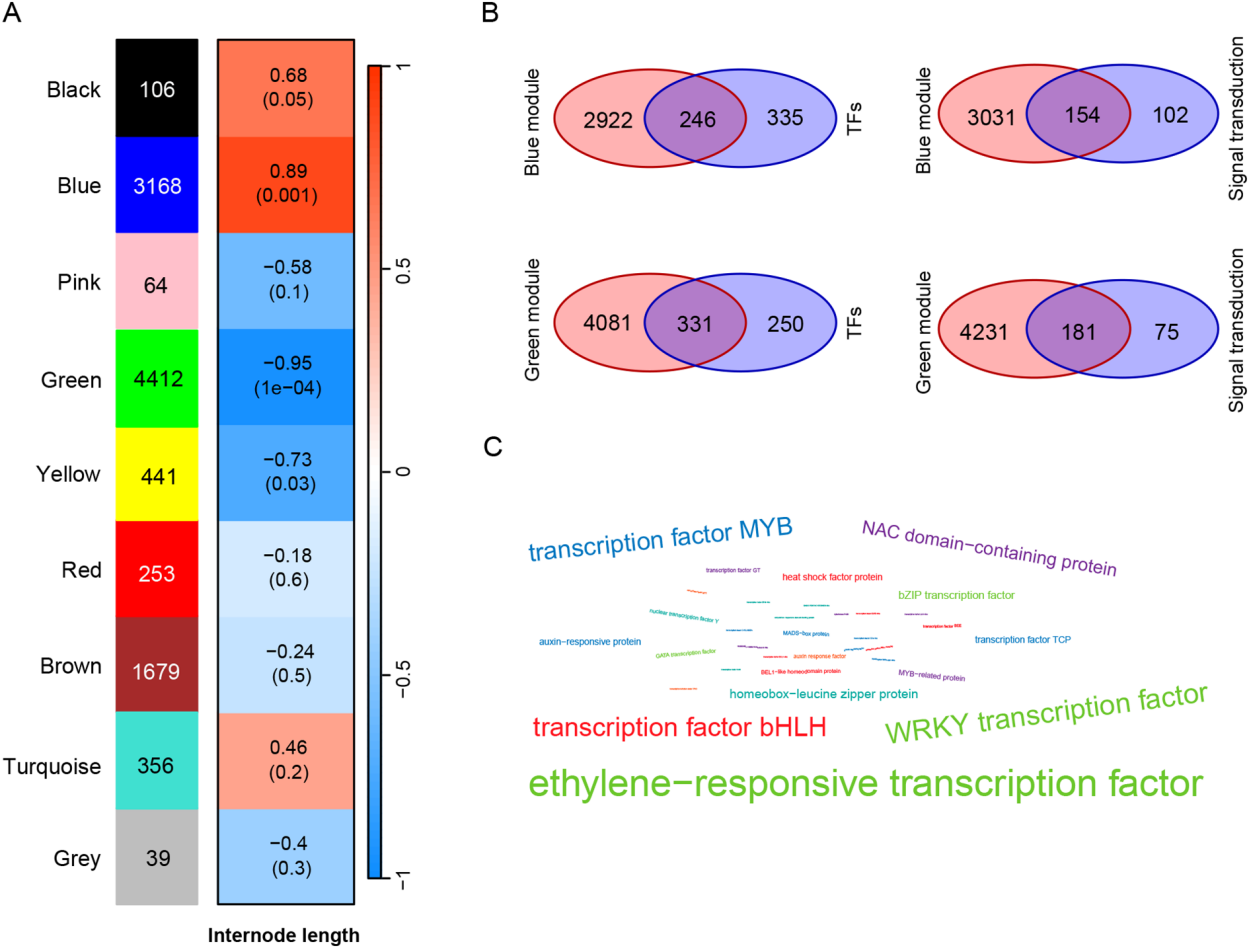
Identification of key genes related to internode length. **(A)** WGCNA analysis of inter-node length and DEGs. The number of genes in each module is listed on the left. The red and blue boxes represent the positive and negative correlations between DEGs and internode length, respectively; **(B)** Venn diagram analysis of DEGs in enrichment pathways and DEGs in blue and green modules; **(C)** Word cloud map of transcription factors in DEGs. The size of the font represents the frequency of transcription factor occurrence.

To explore the DEGs related to internode length of tea plants, we conducted WGCNA analysis on the DEGs identified in three tea varieties and their internode length. The results showed that 10,518 DEGs were clustered into 9 modules based on their expression patterns (**Figure 4A**), namely Black (106 DEGs), Blue (3168 DEGs), Pink (64 DEGs), Green (4412 DEGs), Yellow (441 DEGs), Red (253 DEGs), Brown (1679 DEGs), Turquoise (356 DEGs) and Grey (39 DEGs). In order to further identify DEGs related to stem internode length, we set a screening criterion and considered modules with | r | > 0.8 and P < 0.01 in the correlation analysis results as modules significantly correlated with internode length phenotype. According to this standard, we identified a blue module (r = 0.89, P = 0.001) significantly positively correlated with stem internode length and a green module (r = -0.95, P= 1e-4) significantly negatively correlated with this phenotype. Interestingly, DEGs positively and negatively correlated with internode length were identified in the 9 modules, indicating that gene regulation of tea stem internode length is influenced by both positive and negative factors.

Through the enrichment analysis of DEGs, it was found that signal transduction and transcription factors may play a role in regulating stem elongation. In addition, gene modules significantly positively and negatively correlated with internode length were identified through WGCNA analysis. To further screen for key candidate genes, we performed Venn diagram analysis on the WGCNA results and enrichment results. There are a total of 3168 DEGs in the blue module positively correlated with stem length, and 581 DEGs in the enriched transcription factor pathway, of which 246 are significantly positively correlated with stem length (**Figure 4B**). There are 256 DEGs enriched in the signal transduction pathway, and 154 DEGs intersect with the blue module. Using the same method, the DEGs negatively correlated with stem length in the green module were intersected with DEGs in transcription factors and signal transduction pathways, and 331 transcription factors negatively correlated with stem length and 181 signal transduction genes negatively correlated with stem length were identified. Using word cloud analysis to analyze these transcription factors related to stem length, it was found that these transcription factors are mainly ethylene−responsive transcription factor (ERR), WRKY transcription factor, transcription factor bHLH, transcription factor MYB, and NAC domain−containing protein (**Figure 4C**).

**Figure 5:**
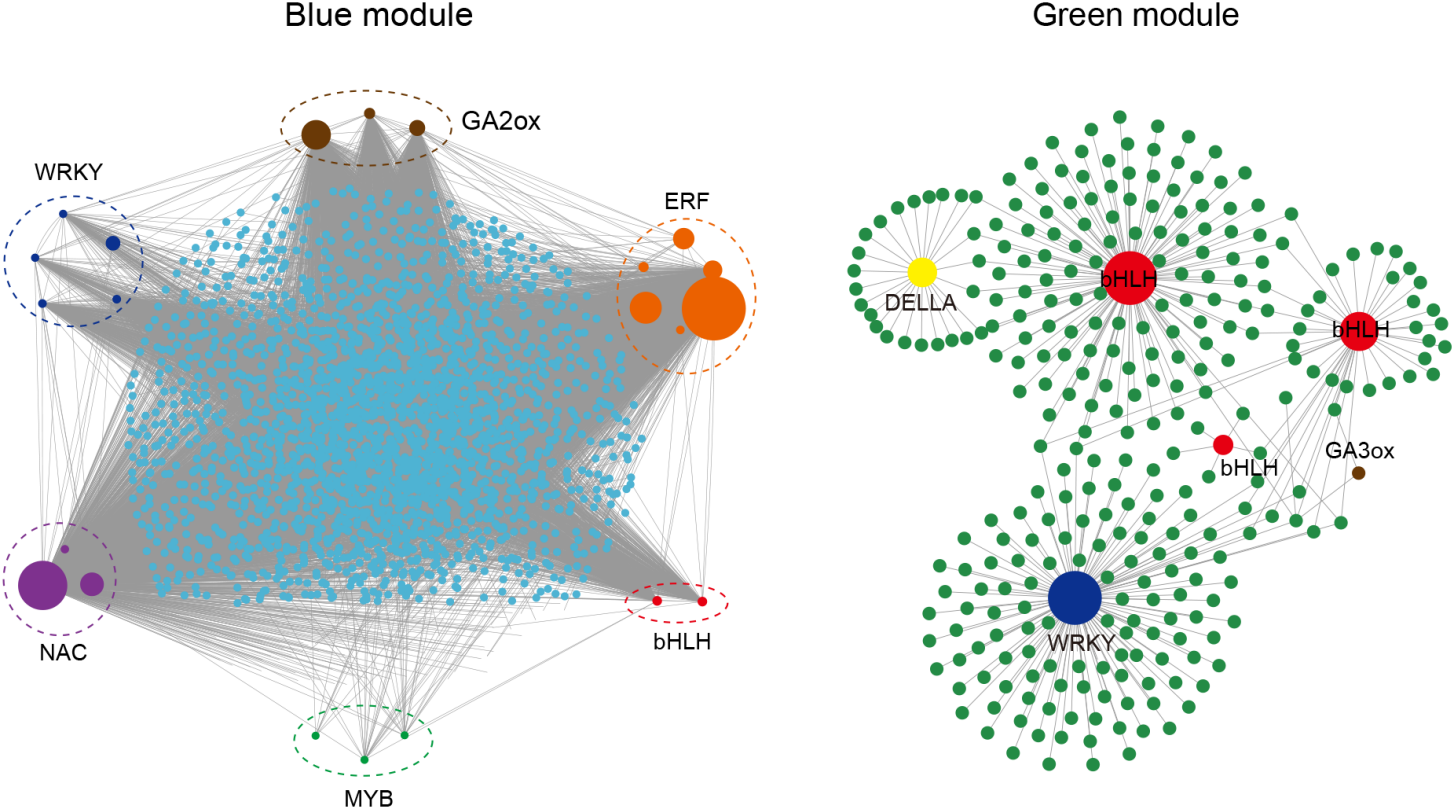
Co-expression network of genes related to internode length constructed by Cytoscape. Different colored circles represent different gene categories. The size of the circle represents the number of genes connected to the node.

These identified candidate genes were used as nodes to construct a co expression network of genes related to stem internode length. In the blue module, a total of 22 candidate genes related to stem length were identified, and these candidate genes were classified into 6 categories according to their gene annotations. Among them, there were 5 types of transcription factors, namely 5 *WRKY*, 6 *ERF*, 3 *NAC*, 2 *bHLH*, and 3 *MYB*. In addition, 3 gibberellin 2-oxidase genes (*GA2ox*) in gibberellin metabolic pathways were identified (**Figure 5**). Based on the number of candidate gene nodes, they are represented by circles of different sizes. The information of all candidate genes is listed in **Supplementary Table S4**. For example, in the blue module, *ERF* (CsQC014983) has a maximum of 2683 connections with other genes, while *MYB* (CsQC027741) has a minimum of 13 connections with other genes (**Supplementary Table S4**). In the green module, a total of 6 candidate genes related to stem length were identified, including 3 *bHLH* transcription factors, 1 WRKY transcription factor, 1 DELLA protein, and 1 gibberellin 3-oxidase genes (*GA3ox*) gene. A total of 28 hub genes were identified in two modules, which may play a positive or negative role in regulating tea stem elongation.

### 3.5 Expression patterns of key genes in three varieties

**Figure 6:**
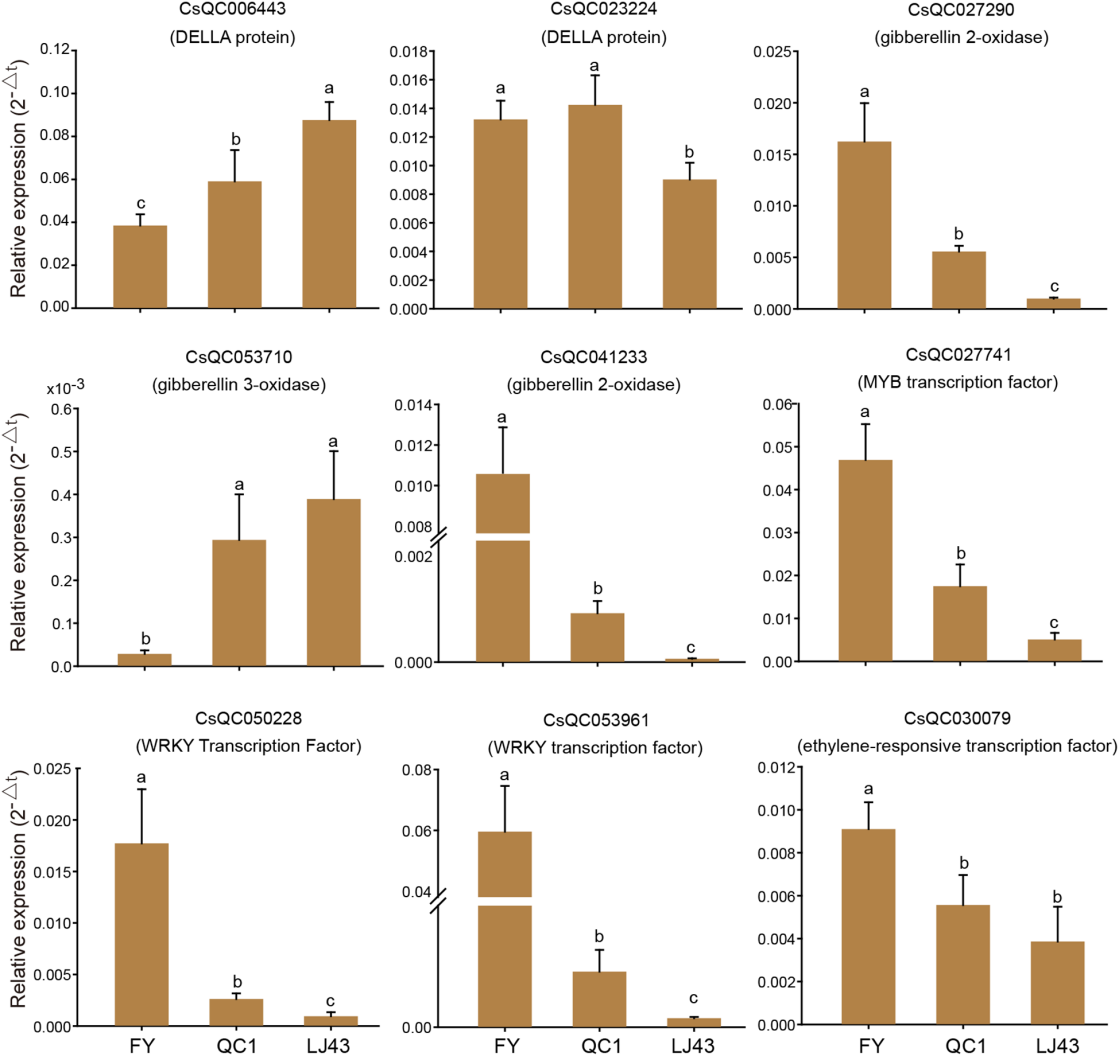
qRT-PCR analysis of key candidate genes related to internode length. The gene ID and annotation are displayed above the bar chart. The lowercase letters a–c represent significant differences at the P < 0.05 level.

The expression patterns of candidate genes related to internode length in three tea varieties were analyzed by qRT-PCR. The results showed that the selected 9 genes were differentially expressed in all three varieties (**Figure 6**). The expression level of one DELLA protein (CsQC006443) gradually increased in FY, QC1, and LJ43, and there were significant differences among the three varieties. This change pattern is negatively correlated with the internode length of the three varieties. Another DELLA protein (CsQC023224) is highly expressed in FY and QC1, while its expression level is lower in LJ43. The expression trends of two *GA2ox* genes (CsQC027290 and CsQC041233) in the three varieties are consistent, with high expression in FY, followed by QC1 with the lowest expression level in LJ43. The expression trend of *GA3ox* (CsQC053710) is opposite to that of GA2ox, with gradually increasing expression levels in FY, QC1, and LJ43. The expression patterns of *MYB* (CsQC027741), *ERF* (CsQC030079), and two *WRKY* transcription factors (CsQC050228 and CsQC053961) in the three varieties are consistent, with the highest expression in FY followed by QC1, and the lowest expression level in LJ43. The expression patterns of these four transcription factors in the three varieties are significantly positively correlated with their stem internode length, which may play a role in regulating stem elongation.

## 4 Discussion

Stem internode length is one of the key traits regulating plant structure, which can affect the light energy utilization efficiency of plants and have an impact on their yield and environmental adaptability (Zhao et al., 2021; Tan et al., 2024). In tea plants, internode length is an important indicator that affects their harvesting efficiency (Luo et al., 2022, 2023). Therefore, selecting tea varieties with appropriate internode length and exploring the molecular regulation mechanism of internode length are of great significance for improving the efficiency of tea plantation harvesting. In this study, we characterized internode length variation among FY, QC1, and LJ43 tea varieties. Compared with LJ43, both QC1 and FY have longer internode lengths, and internode length of FY is significantly longer than that of QC1. This result provides good materials for exploring the mechanism of stem growth and its suitability for mechanical harvesting. In addition, there have been reports on the molecular mechanisms of internode length in other crops, with genes related to the gibberellin pathway and transcription factor regulation being the most common (Ding et al., 2021; Yan et al., 2022). This study identified 28 candidate genes, including *GA3ox*, *DELLA*, *WRKY*, *MYB* etc., that may regulate internode length through transcriptome sequencing combined with DEGs, enrichment analysis, and WGCNA. Compared with previous studies, we identified genes related to stem length at the whole genome level and identified key hormones and transcriptional regulatory metabolic pathways associated with tea plant stem length, proposing potential molecular mechanisms for regulating tea plant stem length.

*GA2ox*, *GA3ox* and *GA20ox* are key genes in the GA synthesis pathway, affecting the content of gibberellin in plants and regulating internode length (Wu et al., 2023). In addition, the DELLA protein in GA signal transduction is also related to the regulation of internode length by GA (Wu et al., 2021). In this study, genes *GA2ox*, *GA3ox*, and *DELLA* were also identified, which is similar to previous reports and suggests that they may regulate the elongation of tea plant stem internodes. The *GA3ox* and *GA20ox* genes promote the synthesis of active GA, which in turn promotes the elongation of plant internode length (Yu et al., 2021; Paciorek et al., 2022). GA2 promotes the synthesis of inactive GA, and DELLA protein is an inhibitory factor of GA signaling pathway, which generally has a negative regulatory effect on internode length (Shi et al., 2024). qRT-PCR showed that the expression of *DELLA* gene gradually increased in FY, QC1, and LJ43, and was significantly negatively correlated with its internode length. This also suggests that DELLA protein may have an inhibitory effect on tea stem elongation. The expression patterns of *GA2ox* and *GA3ox* genes are positively and negatively correlated with internode length, respectively. This may be due to the dynamic regulation changes in material growth status, environment, and GA. Based on the current results, we have identified them as key genes regulating internode length in tea plants. However, whether they have a positive or negative regulatory effect needs to be verified through transgenic methods using a stable genetic transformation system in the future.

In addition to the gibberellin pathway, the regulation of transcription factors is also involved in the elongation process of plant stems. A R2R3-MYB transcription factor MYB112 has been found to promote elongation of hypocotyls in *Arabidopsis* (Cai et al., 2023). We found that the expression levels of *MYB*, *WRKY*, and *ERF* gradually decreased in FY, QC1, and LJ43, and were significantly positively correlated with their internode length. This suggests that these transcription factors may promote the elongation of tea tree stems. However, further experimental verification is needed for its functionality, and after determining its effect on internode elongation, its target genes will be further identified, and the regulatory network of transcription factors target genes internode length will be analyzed. In addition, studies have found that ERF and WRKY transcription factors can regulate the expression of *GA3ox*, *GA20ox*, and *GA2xox* in the GA synthesis pathway in *Arabidopsis* and rice, thereby regulating stem development (Zhou et al., 2016; Wei et al., 2021). Previous studies on internode length in tea plants have mostly focused on the GA metabolic pathway. This study identified candidate genes for *MYB*, *WRKY*, and *ERF* transcription factors in transcriptional regulation, and we found that a positive or negative correlation between the identified transcription factors and genes in the GA pathway, which may be able to regulate the expression of these genes.

## 5 Conclusion

In summary, our study identified significant differences in internode length among three tea plant varieties. Through RNA seq, DEGs analysis, enrichment analysis, and association analysis with internode length, it was found that the regulation of transcription factors and the GA pathway are the main pathways affecting internode length in tea plants. Afterwards, key genes that may regulate the internode length of tea plants were identified through these pathways, such as *DELLA*, *GA2ox*, *GA3ox*, *WRKY* and *ERF*. These findings provide insights into the molecular mechanisms of stem elongation in tea plants and lay the foundation for the breeding of tea tree varieties suitable for machine harvesting.

## Data Availability

The datasets presented in this study can be found in online repositories. The names of the repository/repositories and accession number(s) can be found below: https://ngdc.cncb.ac.cn, PRJCA036838.
